# Design and Application of Quadrature Compensation Patterns in Bulk Silicon Micro-Gyroscopes

**DOI:** 10.3390/s141120419

**Published:** 2014-10-29

**Authors:** Yunfang Ni, Hongsheng Li, Libin Huang

**Affiliations:** 1 School of Instrument Science and Engineering, Southeast University, Nanjing 210096, China; E-Mails: niyunfang@126.com (Y.N.); huanglibin@seu.edu.cn (L.H.); 2 Key Laboratory of Micro-Inertial Instrument and Advanced Navigation Technology of Ministry of Education, Southeast University, Nanjing 210096, China

**Keywords:** MEMS, silicon micro-gyroscope, quadrature compensation, mechanical design

## Abstract

This paper focuses on the detailed design issues of a peculiar quadrature reduction method named system stiffness matrix diagonalization, whose key technology is the design and application of quadrature compensation patterns. For bulk silicon micro-gyroscopes, a complete design and application case was presented. The compensation principle was described first. In the mechanical design, four types of basic structure units were presented to obtain the basic compensation function. A novel layout design was proposed to eliminate the additional disturbing static forces and torques. Parameter optimization was carried out to maximize the available compensation capability in a limited layout area. Two types of voltage loading methods were presented. Their influences on the sense mode dynamics were analyzed. The proposed design was applied on a dual-mass silicon micro-gyroscope developed in our laboratory. The theoretical compensation capability of a quadrature equivalent angular rate no more than 412 °/s was designed. In experiments, an actual quadrature equivalent angular rate of 357 °/s was compensated successfully. The actual compensation voltages were a little larger than the theoretical ones. The correctness of the design and the theoretical analyses was verified. They can be commonly used in planar linear vibratory silicon micro-gyroscopes for quadrature compensation purpose.

## 1. Introduction

Silicon micro-gyroscopes have achieved rapid development in the past several decades. In contrast with their traditional counterparts, silicon micro-gyroscopes have the advantages of small size, reduced power consumption and batch fabrication, *etc*. They are nowadays widely used in commercial and military fields, such as consumer electronics, automobile industry, aerospace navigation, weapons and military supplies, *etc*.

The silicon micro-gyroscope is a kind of Coriolis vibratory gyroscope. It commonly works based on the Coriolis-effect-induced energy transmission between two orthogonal vibration modes, namely drive mode and sense mode. Generally, a Coriolis mass is actuated into resonant vibration with constant amplitude in the drive direction. When an angular rate is applied, a Coriolis-effect-induced Coriolis force will appear in the sense direction, which is proportional to the drive-mode velocity as well as the value of the input angular rate. Actuated by this Coriolis force, a Coriolis vibration response exists in the sense direction. Therefore, through the detection of the sense-mode position, the value of the input angular rate can be obtained.

However, due to non-ideal factors such as fabrication imperfections, other coupling mechanisms also exist between these two vibration modes, introducing bias into the gyroscope output. The commonly seen coupling mechanisms are elastic coupling, viscous coupling and electrostatic coupling, among which elastic coupling is the largest in magnitude most of the time. Elastic coupling is mainly caused by the anisoelasticity either existing in the suspension elements themselves or introduced by fabrication imperfections, that is, an off-diagonal coupling stiffness often exists in the mechanical stiffness matrix of the micro-gyroscope structure. Because of this mechanical coupling stiffness, a quadrature force will appear in the sense direction, which is proportional to the drive-mode position. Therefore, quadrature vibration response also exists in the sense direction and will mix into the gyroscope output if it is not thoroughly distinguished from the Coriolis vibration response. In comparison with the amplitude of the Coriolis response, that of the quadrature response is often considerably large. Hence, quadrature reduction has become one of the primary issues in the design of high-performance silicon micro-gyroscopes.

So far various quadrature reduction methods have been reported [1,2]. They can be classified into two categories: signal nulling and movement correction. In the signal nulling category, there are mainly two specific methods:

(1) Synchronous demodulation. During signal processing, the Coriolis signal is distinguished from the quadrature signal through synchronous demodulation according to their 90° phase difference.
(2) Targeted signal injection. A compensation signal with the same amplitude but opposite phase is generated and injected into the input of the detection circuits to counteract the quadrature signal.

In the movement correction category, there are mainly three specific methods:

(1) Mechanical stiffness matrix diagonalization. The off-diagonal entry of the mechanical stiffness matrix is eradicated by post-fabrication trimming, e.g., laser trimming, on each individual microstructure. Once the mechanical stiffness matrix becomes diagonal, the quadrature vibration response no longer exists.
(2) System stiffness matrix diagonalization. An electrostatic stiffness matrix is constructed with purposefully designed quadrature compensation patterns and properly applied DC voltages. The system stiffness matrix is then the sum of the naturally existed mechanical stiffness matrix and the artificially introduced electrostatic stiffness matrix. When the off-diagonal entry of the electrostatic stiffness matrix has the same value but opposite sign with that of the mechanical stiffness matrix, the system stiffness matrix would be diagonalized and the quadrature vibration response no longer exists.
(3) Force feedback balancing. A balancing force that has the same value but opposite phase with the quadrature force is generated by the closed-loop force feedback circuits to cancel out the quadrature vibration response.

Obviously, among these quadrature reduction methods, the movement correction category is superior to the signal nulling category because it eliminates the quadrature vibration response at the source. Furthermore, among the three specific movement correction methods, system stiffness matrix diagonalization is superior to the other two. Compared with mechanical stiffness matrix diagonalization, it costs rather less and is much easier to control. Compared with force feedback balancing, it extracts the frequency and phase information mechanically from the drive-mode position, hence it avoids the need for precise frequency and phase control.

The core idea of system stiffness matrix diagonalization is the modification of the system stiffness distribution, that is, a proper distribution of electrostatic stiffness is introduced to balance the non-ideal distribution of mechanical stiffness. This method has already been employed in vibrating ring or cup gyroscopes for a long time. In 1996, Clark first introduced its application in surface silicon micro-gyroscopes [3]. However, its application in bulk silicon micro-gyroscopes has not been reported until the past several years [4–7]. Though several successful application examples have been reported, the concrete design issues have not been discussed in detail yet. In this paper, we focus on the detailed design issues of this method.

The rest of this paper is organized as follows: Section 2 describes the compensation principle of system stiffness matrix diagonalization based on the introduction of the arising mechanism and the characteristics of the quadrature vibration response. Section 3 discusses the mechanical design issues of system stiffness matrix diagonalization, including the classification of the basic structural units, the layout design of quadrature compensating patterns and the optimization of key structural parameters. Section 4 discusses the voltage loading methods of system stiffness matrix diagonalization. Two methods were proposed and their influences on the sense-mode dynamics were analyzed. Section 5 presents an actual application of the design theories on a dual-mass silicon micro-gyroscope developed in our laboratory. Section 6 provides the experimental results on a packaged silicon micro-gyroscope prototype. The correctness of the design and the theoretical analyses was verified. Section 7 concludes the whole paper.

## 2. Compensation Principle

Quadrature vibration response is mainly caused by the off-diagonal entry of the mechanical stiffness matrix [2]. Considering this mechanical coupling stiffness, the dynamic equations of a linear vibratory silicon micro-gyroscope can be expressed as:

(1) $$\left[\begin{array}{cc} m_{c} & 0\\ 0 & m_{c} \end{array}\right]\;\left[\begin{array}{c} \ddot{x}\\ \ddot{y} \end{array}\right]+\left[\begin{array}{cc} c_{x} & 0\\ 0 & c_{y} \end{array}\right]\;\left[\begin{array}{c} \dot{x}\\ \dot{y} \end{array}\right]+\left[\begin{array}{cc} k_{x} & k_{xy}\\ k_{yx} & k_{y} \end{array}\right]\;\left[\begin{array}{c} x\\ y \end{array}\right]=\left[\begin{array}{c} F_{d}\\ F_{c} \end{array}\right]$$

where x, y represent the mass position in drive and sense direction; m_c_ represents the Coriolis mass; c_x_, c_y_ represent the damping along X-axis and Y-axis; k_x_, k_y_ represent the stiffness along *X*-axis and *Y*-axis; k_xy_ and k_yx_ are the mechanical coupling stiffness between drive and sense directions which bring about the quadrature vibration response; F_d_ is the driving force and F_c_ is the Coriolis force.

Considering the common seen condition that the natural frequencies of the drive mode and the sense mode are mismatched to ensure a certain open-loop bandwidth, the mass position in sense direction would be much smaller than that in drive direction, *i.e.*, y ≪ x. Hence, the influence of k_xy_ and y on the solution of x was ignored in the following analysis. We defined the quadrature force as F_q_. Then the dynamic equation in drive direction and that in sense direction can be expressed as:

(2) $$m_{c}\ddot{x}+c_{x}\dot{x}+k_{x}x\approx F_{d}$$

(3) $$m_{c}\ddot{y}+c_{y}\dot{y}+k_{y}y=F_{c}+F_{q}$$

The Coriolis force F_c_ and the quadrature force F_q_ can be expressed as:

(4) $$\begin{array}{ccc} F_{c}=-2m_{c}\Omega_{z}\dot{x} & , & F_{q}=-k_{yx}x \end{array}$$

where Ω_z_ is the value of the input angular rate about *Z*-axis. The Coriolis mass is usually actuated into resonant vibration with constant amplitude in drive direction. Therefore, the driving force F_d_ and the corresponding solution of drive-mode position x can be expressed as:

(5) $$\begin{array}{ccc} F_{d}=F_{0}\sin\left(\omega_{d}t\right) & , & x=A_{d}\sin\left(\omega_{d}t-90{}^{\circ}\right) \end{array}$$

where F_0_ is the amplitude of the driving force, ω_d_ is the natural frequency of the drive mode and A_d_ is the amplitude of the drive-mode position. In Equation (4), it is obvious that Coriolis force F_c_ is proportional to the drive-mode velocity and quadrature force F_q_ is proportional to the drive-mode position. With the same frequency they have a 90° phase difference with each other. With Equation (5), they can be further expressed as:

(6) $$\begin{array}{ccc} F_{c}=-2m_{c}\Omega_{z}\omega_{d}A_{d}\sin\left(\omega_{d}t\right) & , & F_{q}=-k_{yx}A_{d}\sin\left(\omega_{d}t-90{}^{\circ}\right) \end{array}$$

We defined the sense-mode position caused by the Coriolis force F_c_ as Coriolis vibration response y_c_, and the sense-mode position caused by the quadrature force F_q_ as quadrature vibration response y_q_. With Equations (3) and (6), the solutions of them could be obtained as:

(7) $$y_{c}=\frac{-2\Omega_{z}\omega_{d}A_{d}}{\sqrt{{\left(\omega_{s}^{2}-\omega_{d}^{2}\right)}^{2}+{\left(\frac{\omega_{s}\omega_{d}}{Q_{s}}\right)}^{2}}}\sin\left(\omega_{d}t+\phi \right),y_{q}=\frac{-k_{yx}A_{d}/m_{c}}{\sqrt{{\left(\omega_{s}^{2}-\omega_{d}^{2}\right)}^{2}+{\left(\frac{\omega_{s}\omega_{d}}{Q_{s}}\right)}^{2}}}\sin\left(\omega_{d}t-90{}^{\circ}+\phi \right)$$

where ω_s_ is the natural frequency of the sense mode, Q_s_ is the quality factor of the sense mode and φ is the phase shift, which can be expressed as:

(8) $$\phi =-\arctan\frac{\omega_{s}\omega_{d}/Q_{s}}{\omega_{s}^{2}-\omega_{d}^{2}}$$

From Equations (6) and (7), it can be found that Coriolis vibration response y_c_ and quadrature vibration response y_q_ have the same relative relationship as that which exists between Coriolis force F_c_ and quadrature force F_q_, that is, y_c_ and y_q_ still have the same frequency and a 90° phase difference with each other. It is obvious that though y_c_ and y_q_ are both affected by the quality factor and the natural frequencies, the relative relationship between them is independent of the sense-mode dynamics. Therefore, the quadrature equivalent angular rate Ω_q_ of the quadrature vibration response y_q_ can be expressed as:

(9) $$\Omega_{q}=\frac{\left|y_{q}\right|}{\left|y_{c}\right|/\Omega_{z}}=\frac{\left|k_{yx}\right|}{2m_{c}\omega_{d}}$$

The key technology of system stiffness matrix diagonalization is the proper construction of an electrostatic stiffness matrix. With **K**_M_, **K**_E_ and **K**_S_ denoting the mechanical, electrostatic and system stiffness matrix respectively, the compensation principle of system stiffness matrix diagonalization can be described as:

(10) $$\overset{{\text{K}}_{M}}{\overset{︷}{\left[\begin{array}{cc} k_{x} & k_{xy}\\ k_{yx} & k_{y} \end{array}\right]}}+\overset{K_{E}}{\overset{︷}{\left[\begin{array}{cc} k_{ex} & k_{\text{exy}}\\ k_{\text{eyx}} & k_{ey} \end{array}\right]}}=\overset{K_{S}}{\overset{︷}{\left[\begin{array}{cc} k_{x}+k_{ex} & 0\\ 0 & k_{y}+k_{ey} \end{array}\right]}}\Leftrightarrow \{\begin{array}{c} k_{\text{exy}}=-k_{xy}\\ k_{\text{eyx}}=-k_{yx} \end{array}$$

where k_ex_, k_ey_ represent the electrostatic stiffness along *X*-axis and *Y*-axis respectively; k_exy_, k_eyx_ are the electrostatic coupling stiffness between the drive and sense directions contributing to the quadrature compensation. When the electrostatic coupling stiffness has the same value but opposite sign as the mechanical coupling stiffness, the system stiffness matrix would be diagonalized and quadrature vibration response no longer exists.

**Figure 1.**
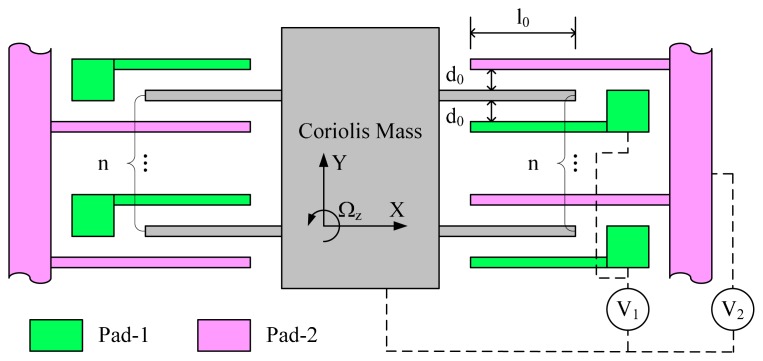
An example of quadrature compensation patterns used in surface silicon micro-gyroscopes.

The electrostatic stiffness matrix could be electromechanically provided by purposefully designed quadrature compensation patterns and properly applied DC voltages. A typical example of quadrature compensation patterns used in surface silicon micro-gyroscopes are shown in Figure 1. It consists of n sets of parallel plate capacitors. DC voltages with different values, V_1_ and V_2_, are applied on Pad-1 and Pad-2 respectively. Considering the common seen condition that the mass position in sense direction is much smaller than the initial gap of the parallel plate capacitors, *i.e.*, y ≪ d_0_, the electrostatic stiffness matrix provided by this quadrature compensation patterns and the DC voltages can be expressed as:

(11) $$K_{E}=\left[\begin{array}{cc} k_{ex} & k_{\text{exy}}\\ k_{\text{eyx}} & k_{ey} \end{array}\right]=\left[\begin{array}{cc} 0 & \frac{n\varepsilon_{0}h_{0}}{d_{0}^{2}}\left(V_{1}^{2}-V_{2}^{2}\right)\\ \frac{n\varepsilon_{0}h_{0}}{d_{0}^{2}}\left(V_{1}^{2}-V_{2}^{2}\right) & -\frac{2n\varepsilon_{0}h_{0}l_{0}}{d_{0}^{3}}\left(V_{1}^{2}+V_{2}^{2}\right) \end{array}\right]$$

where ε_0_ is the permittivity of vacuum, h_0_ is the thickness of the structure and l_0_ is the initial overlap length of the parallel plate capacitors.

It can be seen from Equation (11) that an electrostatic coupling stiffness is provided, whose value and sign can be adjusted by the capacitor parameters and the DC voltages. For an actual silicon micro-gyroscope, through measuring the amplitude and phase of the quadrature vibration response, and with the help of Equation (9), the value and sign of the mechanical coupling stiffness could be obtained. With the quadrature compensation patterns and the regulation of the DC voltages, the electrostatic coupling stiffness can be made to have the same value but opposite sign with the mechanical coupling stiffness. Once this condition is met, system stiffness matrix diagonalization is realized and quadrature vibration response no longer exists.

## 3. Design and Analysis of Quadrature Compensation Patterns

The quadrature compensation patterns illustrated in Figure 1 are more suitable in surface silicon micro-gyroscopes. In this type of quadrature compensation patterns, the anchoring area of Pad-2 is continuous but that of Pad-1 is discrete, that is, each Pad-1 needs an individual anchoring area. That would be rather area consuming in bulk silicon micro-fabrication because each anchoring area should be large enough to ensure the bonding reliability. Hence, the number of the capacitor sets and consequently the compensation capability would be limited. Therefore, for quadrature compensation patterns in bulk silicon micro-gyroscopes, comb capacitors are more commonly used than parallel plate capacitors. With the help of an assistant larger gap which we defined as p times larger than the initial gap d_0_, *i.e.*, pd_0_, the anchoring area of all pads would be continuous. Though the electrostatic coupling stiffness provided by a single set of comb capacitors is a little smaller than that of parallel plate capacitors, the available number of the comb capacitor sets would be much more than that of the parallel plate capacitor sets in a limited layout area. Therefore, in bulk silicon micro-gyroscopes, quadrature compensation patterns which consist of comb capacitors can provide a better overall compensation capability than those consist of parallel plate capacitors. In the following discussion, we focus on the design issues of quadrature compensation patterns which consist of comb capacitors in bulk silicon micro-gyroscopes.

### 3.1. Basic Structural Units

**Figure 2.**
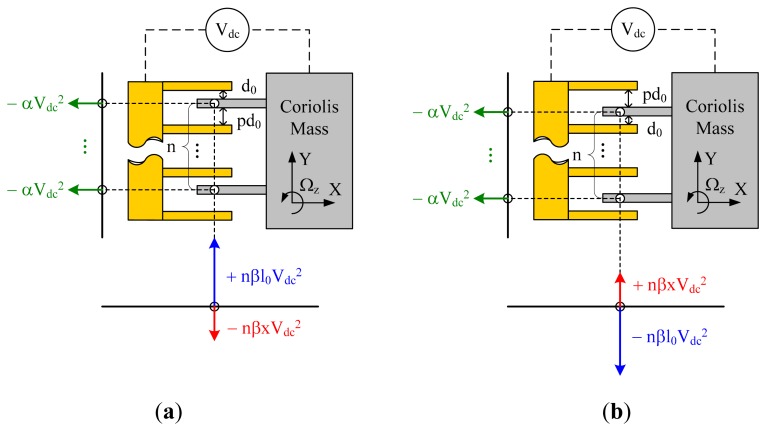

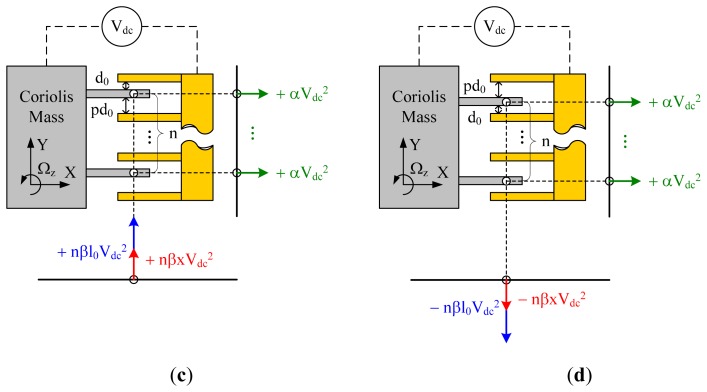
(**a**) Basic structural unit Type-A; (**b**) Basic structural unit Type-B; (**c**) Basic structural unit Type-C; (**d**) Basic structural unit Type-D.

According to the relative position between the Coriolis mass and the stationary pads, there are four types of basic structural units in the quadrature compensation patterns. The structural schematics of them along with the electrostatic forces they generated in drive and sense directions are shown in Figure 2. V_dc_ is the applied DC voltage. For the convenience of analysis, we defined three structural constants, α, β and γ, as follows:

(12) $$\alpha =\frac{\varepsilon_{0}h_{0}}{2d_{0}}\left(1+\frac{1}{p}\right),\beta =\frac{\varepsilon_{0}h_{0}}{2d_{0}^{2}}\left(1-\frac{1}{p^{2}}\right),\gamma =\frac{\varepsilon_{0}h_{0}}{d_{0}^{3}}\left(1+\frac{1}{p^{3}}\right)$$

Identified by four different subscripts, A, B, C and D, the electrostatic forces and electrostatic stiffness matrixes provided by the four types of basic structural units can be expressed as:

(13) $$F_{E\text{A}}=\left[\begin{array}{c} F_{ex}\\ F_{ey} \end{array}\right]=\left[\begin{array}{c} -n\alpha V_{dc}^{2}\\ +n\beta l_{0}V_{dc}^{2}-n\beta xV_{dc}^{2} \end{array}\right],{\text{K}}_{EA}=\left[\begin{array}{cc} k_{ex} & k_{\text{exy}}\\ k_{\text{eyx}} & k_{ey} \end{array}\right]=\left[\begin{array}{cc} 0 & +n\beta V_{dc}^{2}\\ +n\beta V_{dc}^{2} & -n\gamma \left(l_{0}-x\right)V_{dc}^{2} \end{array}\right]$$

(14) $$F_{EB}=\left[\begin{array}{c} F_{ex}\\ F_{ey} \end{array}\right]=\left[\begin{array}{c} -n\alpha V_{dc}^{2}\\ -n\beta l_{0}V_{dc}^{2}+n\beta xV_{dc}^{2} \end{array}\right],K_{EB}=\left[\begin{array}{cc} k_{ex} & k_{\text{exy}}\\ k_{\text{eyx}} & k_{ey} \end{array}\right]=\left[\begin{array}{cc} 0 & -n\beta V_{dc}^{2}\\ -n\beta V_{dc}^{2} & -n\gamma \left(l_{0}-x\right)V_{dc}^{2} \end{array}\right]$$

(15) $$F_{E\text{C}}=\left[\begin{array}{c} F_{ex}\\ F_{ey} \end{array}\right]=\left[\begin{array}{c} +n\alpha V_{dc}^{2}\\ +n\beta l_{0}V_{dc}^{2}+n\beta xV_{dc}^{2} \end{array}\right],{\text{K}}_{EC}=\left[\begin{array}{cc} k_{ex} & k_{\text{exy}}\\ k_{\text{eyx}} & k_{ey} \end{array}\right]=\left[\begin{array}{cc} 0 & -n\beta V_{dc}^{2}\\ -n\beta V_{dc}^{2} & -n\gamma \left(l_{0}+x\right)V_{dc}^{2} \end{array}\right]$$

(16) $$F_{E\text{D}}=\left[\begin{array}{c} F_{ex}\\ F_{ey} \end{array}\right]=\left[\begin{array}{c} +n\alpha V_{dc}^{2}\\ -n\beta l_{0}V_{dc}^{2}-n\beta xV_{dc}^{2} \end{array}\right],{\text{K}}_{ED}=\left[\begin{array}{cc} k_{ex} & k_{\text{exy}}\\ k_{\text{eyx}} & k_{ey} \end{array}\right]=\left[\begin{array}{cc} 0 & +n\beta V_{dc}^{2}\\ +n\beta V_{dc}^{2} & -n\gamma \left(l_{0}+x\right)V_{dc}^{2} \end{array}\right]$$

where **F**_E_ denotes the electrostatic force vector, F_ex_, F_ey_ represent the electrostatic force in drive and in sense direction respectively, n is the number of the comb capacitor sets, l_0_ is the initial overlap length of the combs and x is the drive-mode position of the Coriolis mass.

From Equations (13), (14), (15), and (16), conclusions can be made as follows: in the drive direction, the electrostatic forces are all static forces independent of the overlap length. In the sense direction, each electrostatic force can be seen as the combination of a static force and a dynamic force. The static force is proportional to the initial overlap length l_0_ and the dynamic force is proportional to the drive-mode position x. The orientations of the static forces are determined by the relative position between the Coriolis mass and the stationary pads. They are always attractive and tend to increase the capacitor size. The needed electrostatic coupling stiffness is provided by the dynamic forces. In the four types of basic structural units, Type-A and Type-D generate dynamic forces with a negative orientation in sense direction, providing positive electrostatic coupling stiffness. Type-B and Type-C generate dynamic forces with a positive orientation in sense direction, providing negative electrostatic coupling stiffness.

For an actual silicon micro-gyroscope, the sign of the mechanical coupling stiffness is uncertain due to the uncertainty of the fabrication imperfections. Therefore, to ensure the feasibility of quadrature compensation, at least two types of basic structural units providing electrostatic coupling stiffness with opposite signs are needed in the mechanical design of quadrature compensation patterns.

### 3.2. Layout Design

As discussed previously, among the electrostatic forces generated by the basic structural units, besides the dynamic forces which provide the needed electrostatic coupling stiffness, additional static forces also exist in the drive and sense directions. Moreover, with a lever arm between different basic structural units distributed at different places of the planar micro-gyroscope structure, additional static torques may also exist. These additional static forces and torques would cause a disturbance on the movement of the Coriolis mass. They can be cancelled out by a proper arrangement of the basic structural units on the available layout area of the Coriolis mass, *i.e.*, the layout design of quadrature compensation patterns.

**Figure 3.**
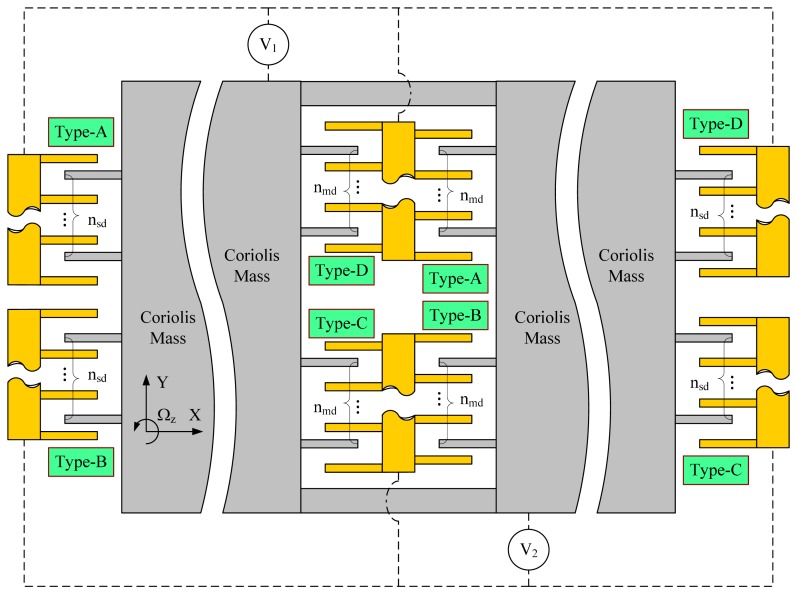
A novel layout design of quadrature compensation patterns.

**Figure 4.**
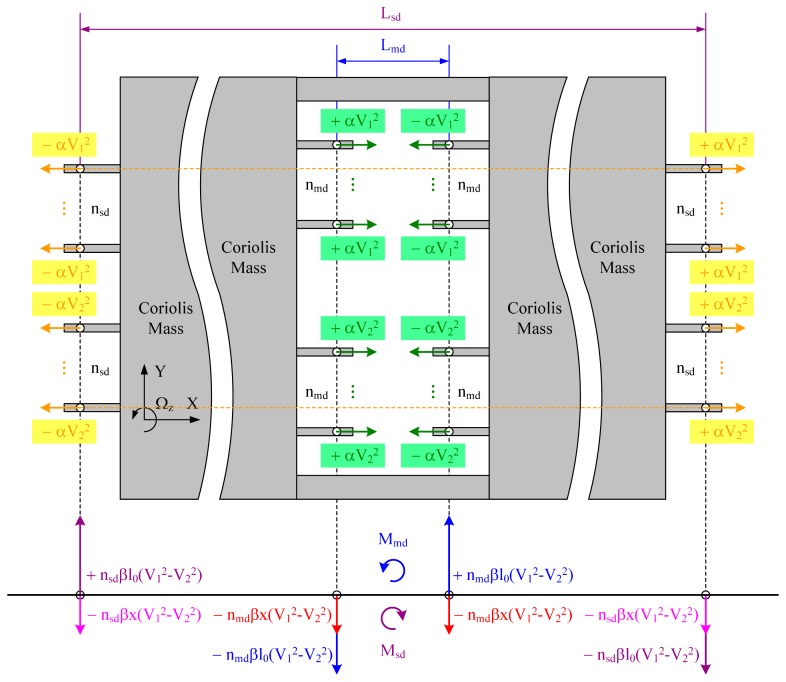
Force and torque distributions of the layout design.

A novel layout design we propose to cancel out the additional static forces and torques is shown in Figure 3. The force and torque distributions of this layout design are shown in Figure 4. As shown in Figure 3, n_md_ sets of basic structural units, including Type-A, Type-B, Type-C and Type-D, are designed in the middle part of the Coriolis mass. All the moveable combs in the four types of basic structural units are axisymmetric. Similarly, n_sd_ sets of basic structural units are designed at the two side parts of the Coriolis mass with all the moveable combs axisymmetric. The basic structural units Type-A and Type-D providing positive electrostatic coupling stiffness are applied with a DC voltage V_1_. The basic structural units Type-B and Type-C providing negative electrostatic coupling stiffness are applied with another DC voltage V_2_.

As shown in Figure 4, in the drive direction, the electrostatic forces generated by Type-A and Type-D cancel each other out, either in the middle part or at the side parts of the Coriolis mass. Likewise, the electrostatic forces generated by Type-B and Type-C in drive direction cancel each other out, either in the middle part or at the side parts of the Coriolis mass.

In the sense direction, as discussed previously, each electrostatic force can be seen as the combination of a static force and a dynamic force. In our analyses, the forces with the same action line in sense direction were added up to get a resultant force. We use the subscripts “sta” and “dyn” to identify the static and dynamic forces, subscripts “md” and “sd” to identify the locations of the basic structural units. As shown in Figure 4, the four static resultant forces can be expressed as:

(17) $$\begin{array}{ll} F_{\text{sta}\_mdAB}=+n_{md}\beta l_{0}\left(V_{1}^{2}-V_{2}^{2}\right), & F_{\text{sta}\_md\text{CD}}=-n_{md}\beta l_{0}\left(V_{1}^{2}-V_{2}^{2}\right) \end{array}$$

(18) $$\begin{array}{cc} F_{\text{sta}\_sdAB}=+n_{sd}\beta l_{0}\left(V_{1}^{2}-V_{2}^{2}\right), & F_{\text{sta}\_sd\text{CD}}=-n_{sd}\beta l_{0}\left(V_{1}^{2}-V_{2}^{2}\right) \end{array}$$

It is obvious that the sum of F_sta_mdAB_ and F_sta_mdCD_ are zero and that of F_sta_sdAB_ and F_sta_sdCD_ are also zero. Hence there is no net static force. However, with a lever arm L_md_ exists between F_sta_mdAB_ and F_sta_mdCD_, a static torque M_md_ turns up. Similarly, with a lever arm L_sd_ exists between F_sta_sdAB_ and F_sta_sdCD_, a static torque M_sd_ turns up. These two static torques can be expressed as:

(19) $$\begin{array}{ccc} M_{md}=+\left[n_{md}\beta l_{0}\left(V_{1}^{2}-V_{2}^{2}\right)\right]\cdot L_{md} & , & M_{sd}=-\left[n_{sd}\beta l_{0}\left(V_{1}^{2}-V_{2}^{2}\right)\right]\cdot L_{sd} \end{array}$$

Aiming to eliminating these static torques, in our layout design, the number of the capacitor sets and the lever arms were designed to follow the following rule:

(20) $$\frac{n_{md}}{n_{sd}}=\frac{L_{sd}}{L_{md}}$$

In this condition, M_md_ and M_sd_ would cancel out with each other. Hence there is no net static torque as well. With Equations (19) and (20), the sum of M_md_ and M_sd_ can be explained as follows:

(21) $$M_{md}+M_{sd}=+\left[{\beta l}^{0}(V_{1}^{2}-V_{2}^{2})\right]\cdot \left(n_{md}L_{md}-n_{sd}L_{sd}\right)=0$$

In conclusion, with the proposed layout design of quadrature compensation patterns, the additional static forces and torques generated by the basic structural units can be cancelled out. Only the useful dynamic forces providing electrostatic coupling stiffness exist in the sense direction.

The four dynamic resultant forces in sense direction can be expressed as:

(22) $$\begin{array}{ccc} F_{\text{dyn}\_mdAB}=-n_{md}\beta x\left(V_{1}^{2}-V_{2}^{2}\right) & , & F_{\text{dyn}\_md\text{CD}}=-n_{md}\beta x\left(V_{1}^{2}-V_{2}^{2}\right) \end{array}$$

(23) $$\begin{array}{ccc} F_{\text{dyn}\_sdAB}=-n_{sd}\beta x\left(V_{1}^{2}-V_{2}^{2}\right) & , & F_{\text{dyn}\_sd\text{CD}}=-n_{sd}\beta x\left(V_{1}^{2}-V_{2}^{2}\right) \end{array}$$

The net dynamic force F_dyn_ and the electrostatic coupling stiffness k_eyx_ it provides can be expressed as:

(24) $$\begin{array}{ccc} F_{\text{dyn}}=-2\left(n_{md}+n_{sd}\right)\beta x\left(V_{1}^{2}-V_{2}^{2}\right) & , & k_{\text{eyx}}=+2\left(n_{md}+n_{sd}\right)\beta \left(V_{1}^{2}-V_{2}^{2}\right) \end{array}$$

With Equation (12), the full expression of the electrostatic coupling stiffness k_eyx_ can be obtained as:

(25) $$k_{\text{eyx}}=+\left(n_{md}+n_{sd}\right)\frac{\varepsilon_{0}h_{0}}{d_{0}^{2}}\left(1-\frac{1}{p^{2}}\right)\left(V_{1}^{2}-V_{2}^{2}\right)$$

With Equations (9), (10) and (25), it can be found that for a certain quadrature equivalent angular rate Ω_q_, the needed value of the DC voltages for quadrature compensation would be:

(26) $$\left|V_{1}^{2}-V_{2}^{2}\right|=\frac{2m_{c}\omega_{d}\cdot \Omega_{q}}{\left(n_{md}+n_{sd}\right)\frac{\varepsilon_{0}h_{0}}{d_{0}^{2}}\left(1-\frac{1}{p^{2}}\right)}$$

The needed relative size of V_1_ and V_2_ is determined by the phase information of the quadrature vibration response.

In addition, it can be found From Equations (13)–(16) that a negative electrostatic stiffness along *Y*-axis is inevitably introduced as long as the comb capacitors have an initial overlap length. The negative electrostatic stiffness k_ey_ introduced by the novel layout design of quadrature compensation patterns shown in Figure 3 can be expressed as:

(27) $$k_{ey}=-\left(n_{md}+n_{sd}\right)\gamma \left(2l_{0}\right)\left(V_{1}^{2}+V_{2}^{2}\right)$$

With Equation (12), the full expression of the negative electrostatic stiffness k_ey_ can be obtained as:

(28) $$k_{ey}=-\left(n_{md}+n_{sd}\right)\frac{2\varepsilon_{0}h_{0}l_{0}}{d_{0}^{3}}\left(1+\frac{1}{p^{3}}\right)\left(V_{1}^{2}+V_{2}^{2}\right)$$

### 3.3. Parameter Optimization

Obviously, the quadrature compensation capability is determined by the value of the electrostatic coupling stiffness provided by the quadrature compensation patterns and the applied DC voltages. Considering the power consumption of a silicon micro-gyroscope, the available values of the DC voltages are generally limited. Therefore, an optimization of the structural parameters is necessary to improve the quadrature compensation capability.

From Equation (25) it can be seen that the main structural parameters affecting the value of the electrostatic coupling stiffness are the thickness h_0_, the initial gap d_0_, the set numbers n_md_, n_sd_ and the gap ratio p (*p* > 1). Among these parameters, h_0_ and d_0_ are often chosen according to the bulk silicon micro-fabrication process. Increasing the gap ratio p can increase the electrostatic coupling stiffness provided by a single set of basic structure unit. However, it would decrease the set numbers n_md_, n_sd_ at the same time. Therefore, a proper design of the gap ratio p is crucial in parameter optimization.

In our layout design shown in Figure 3, the lever arms L_md_, L_sd_ and the set numbers n_md_, n_sd_ follow the design rule shown in Equation (20) and always have the following relationship:

(29) $$\begin{array}{ccc} L_{md}<L_{sd} & , & n_{md}>n_{sd} \end{array}$$

We defined the available length of the Coriolis mass along Y-axis which can be used for the design of quadrature compensation patterns as L_qy_, and the comb width in the basic structural units as w. Then the available set numbers would be:

(30) $$\begin{array}{ccc} n_{md}=\frac{L_{qy}/2-w}{2w+\left(1+p\right)d_{0}} & , & n_{sd}=n_{md}\frac{L_{md}}{L_{sd}} \end{array}$$

With Equations (25) and (30), the available electrostatic coupling stiffness can be expressed as:

(31) $$k_{\text{eyx}}=+\frac{L_{qy}/2-w}{2w+\left(1+p\right)d_{0}}\left(1+\frac{L_{md}}{L_{sd}}\right)\frac{\varepsilon_{0}h_{0}}{d_{0}^{2}}\left(1-\frac{1}{p^{2}}\right)\left(V_{1}^{2}-V_{2}^{2}\right)$$

Therefore, when w, h_0_ and d_0_ have constant values, k_eyx_ and p would have the following relationship:

(32) $$k_{\text{eyx}}\propto \eta =\frac{1-\frac{1}{p^{2}}}{2w+\left(1+p\right)d_{0}}$$

where η is a defined variable identifying how large a k_eyx_ can be obtained with a certain value of p.

**Figure 5.**
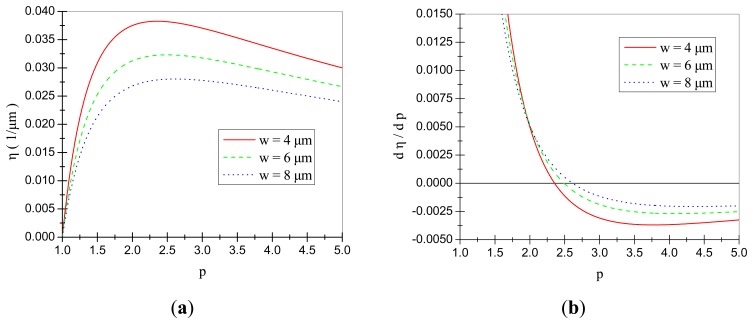
(**a**) Change tendency of η with the variation of p; (**b**) Change tendency of dη/dp with the variation of p.

Considering the bulk silicon micro-fabrication process adopted in our laboratory, the initial gap d_0_ is most commonly chosen as 4 μm. Then with several different values of the comb width w, the influences of the gap ratio p on the value of the variable η are shown in Figure 5. In is obvious that with a proper design of gap ratio p, the variable η and then the electrostatic coupling stiffness k_eyx_ can be maximized within a limited available length L_qy_. When the comb width w was chosen as 4, 6 and 8 μm respectively, the optimal values of p would be 2.36, 2.49 and 2.61.

## 4. Design and Analysis of Voltage Loading Methods

In the process of quadrature compensation, the regulation of the DC voltages relies on the change tendency of the quadrature vibration response y_q_ with the variation of V_1_ and V_2_. The main influence of quadrature compensation on the dynamic equation in sense direction is the introduction of an electrostatic coupling stiffness k_eyx_ and a negative electrostatic stiffness k_ey_. With no input angular rate, the dynamic equation in sense direction during quadrature compensation can be expressed as:

(33) $$m_{c}ÿ+c_{y}ẏ+(k_{y}+k_{ey})y=-(k_{yx}+k_{\text{eyx}})x$$

With Equations (5) and (33), the steady-state solution of the quadrature vibration response y_q_ in the process of quadrature compensation can be expressed as:

(34) $$y_{q}=\frac{-\left(k_{yx}+k_{\text{eyx}}\right)A_{d}/m_{c}}{\sqrt{{\left(\frac{k_{y}+k_{ey}}{m_{c}}-\omega_{d}^{2}\right)}^{2}+\frac{k_{y}+k_{ey}}{m_{c}}{\left(\frac{\omega_{d}}{Q_{s}}\right)}^{2}}}\sin\left(\omega_{d}t-90{}^{\circ}+\phi \right),\phi =-\arctan\left(\frac{\sqrt{\frac{k_{y}+k_{ey}}{m_{c}}}\frac{\omega_{d}}{Q_{s}}}{\frac{k_{y}+k_{ey}}{m_{c}}-\omega_{d}^{2}}\right)$$

For the convenience of analysis, we named the upper half of the quadrature compensation patterns shown in Figure 3 as Group-1. It consists of basic structural units Type-A, Type-D and provides positive electrostatic coupling stiffness. Likewise, we named the lower half of the quadrature compensation patterns shown in Figure 3 as Group-2. It consists of basic structural units Type-B, Type-C and provides negative electrostatic coupling stiffness. Group-1 is applied with DC voltage V_1_ and Group-2 is applied with DC voltage V_2_. Generally, there are two voltage loading methods:

(1) Single group loading. The sign of the mechanical coupling stiffness was firstly obtained through measuring the phase of the quadrature vibration response. Then according to the needed sign of the electrostatic coupling stiffness, positive or negative, only one group of the patterns, Group-1 or Group-2, was applied with a DC voltage. Through the regulation of this DC voltage, V_1_ or V_2_, the amplitude of the quadrature vibration response was reduced to zero. The voltage loading group was chosen manually. Hence this method is suitable for off-line compensation.
(2) Double group loading. Both groups of the patterns, Group-1 and Group-2, were applied with DC voltages, V_1_ and V_2_. The sign of the electrostatic coupling stiffness, positive or negative, was decided by the relative size of V_1_ and V_2_. Through the regulation of |V_1_^2^ − V_2_^2^|, the amplitude of the quadrature vibration response was reduced to zero. Commonly, these two DC voltages were chosen as follows:
  (35) $$\begin{array}{ccc} V_{1}=V_{D}+V_{q}, & V_{2}=V_{D}-V_{q}, & V_{1}^{2}-V_{2}^{2}=4V_{D}V_{q} \end{array}$$

where V_D_ is the preset bias voltage and V_q_ is the regulation voltage. With a constant V_D_, the regulation of V_q_ can change both the sign and the value of the electrostatic coupling stiffness. Hence this method is suitable for on-line compensation.

When single group loading was employed, assuming that the mechanical coupling stiffness was negative, with Equations (24), (27) and (34), the relationship between the amplitude of the quadrature vibration response |y_q_| and the applied DC voltage V_1_ can be expressed as:

(36) $$\left|y_{q}\right|=\frac{\left|-\left|k_{yx}\right|+2\left(n_{md}+n_{sd}\right)\beta \cdot V_{1}^{2}\right|A_{d}/m_{c}}{\sqrt{{\left(\frac{k_{y}-2\left(n_{md}+n_{sd}\right)\gamma l_{0}\cdot V_{1}^{2}}{m_{c}}-\omega_{d}^{2}\right)}^{2}+\frac{k_{y}-2\left(n_{md}+n_{sd}\right)\gamma l_{0}\cdot V_{1}^{2}}{m_{c}}{\left(\frac{\omega_{d}}{Q_{s}}\right)}^{2}}}$$

When double group loading was employed, a constant bias voltage V_D_ was preset. With Equations (24), (27), (34) and (35), the relationship between the amplitude of the quadrature vibration response |y_q_| and the regulation voltage V_q_ can be expressed as:

(37) $$\left|y_{q}\right|=\frac{\left|-\left|k_{yx}\right|+2\left(n_{md}+n_{sd}\right)\beta \cdot 4V_{D}V_{q}\right|A_{d}/m_{c}}{\sqrt{{\left(\frac{k_{y}-2\left(n_{md}+n_{sd}\right)\gamma l_{0}\cdot 2\left(V_{D}^{2}+V_{q}^{2}\right)}{m_{c}}-\omega_{d}^{2}\right)}^{2}+\frac{k_{y}-2\left(n_{md}+n_{sd}\right)\gamma l_{0}\cdot 2\left(V_{D}^{2}+V_{q}^{2}\right)}{m_{c}}{\left(\frac{\omega_{d}}{Q_{s}}\right)}^{2}}}$$

From Equation (36), it can be found that when single group loading was employed, |y_q_| has a non-linear relationship with V_1_. The natural frequency of the sense mode was directly affected by V_1_. It would change a lot in the process of quadrature compensation. From Equation (37), it can be found that when double group loading was employed, if the condition of V_D_ ≫ V_q_ was met, the influence of V_q_ on the natural frequency of the sense mode can be alleviated greatly and |y_q_| would have an approximately linear relationship with V_q_. Therefore, when a smaller change of the natural frequency or a linear relationship between the output and input signals was preferred in the process of quadrature compensation, double group loading would be superior to single group loading.

No matter which voltage loading method was employed, the need quadrature compensation voltage is the one that makes the amplitude of the quadrature vibration response |yq| to be zero. When a voltage smaller than that one was applied, |yq| would be nonzero and the quadrature vibration response would be in the state of undercompensation. When a voltage larger than that one was applied, |y_q_| would also be nonzero and the quadrature vibration response would be in the state of overcompensation.

## 5. Application Example

**Figure 6.**
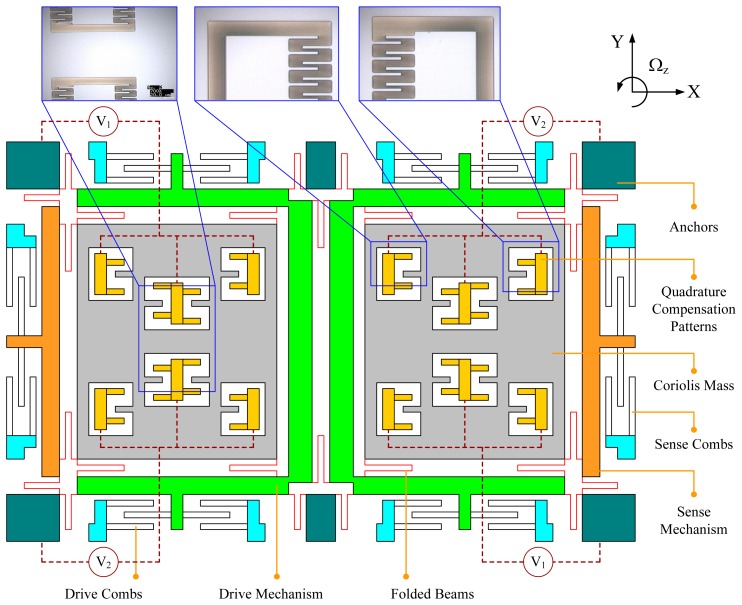
Structural schematic of the dual-mass silicon micro-gyroscope with quadrature compensation patterns.

**Figure 7.**
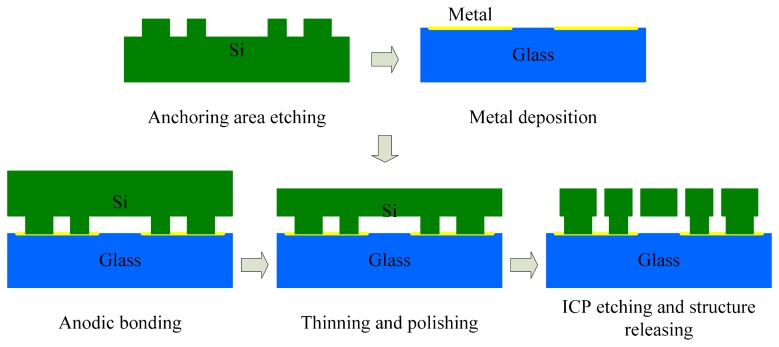
Fabrication process of DDSOG (Deep Dry Silicon on Glass).

The proposed design of quadrature compensation patterns shown in Figure 3 was applied on a dual-mass silicon micro-gyroscope developed in our laboratory. The structural schematic of this dual-mass silicon micro-gyroscope along with three local SEM photos of the basic structural units are shown in Figure 6. The structure of the dual-mass silicon micro-gyroscope is centrosymmetric. Either the left part or the right part of it can be seen as a full-decoupled single-mass silicon micro-gyroscope. The mechanical coupling of these two parts in the drive direction was realized by two folded beams designed between the left and right drive mechanisms. In operation, the left and right parts of this dual-mass silicon micro-gyroscope would vibrate in anti-phase mode and the sense-mode position signal would be obtained differentially. The mechanical structure of this micro-gyroscope was fabricated on (100) silicon wafer and a bulk silicon micro-fabrication process named Deep Dry Silicon on Glass (DDSOG) was adopted. The procedure of this fabrication process is shown in Figure 7.

The main design parameters are shown in Table 1. With Equation (26) it can be found that when the natural frequency of the drive mode is about 4 kHz and the DC voltage applied on a single pattern group is no more than 12 V, the compensation of a quadrature equivalent angular rate Ω_q_ no more than 412 °/s can be realized theoretically by the proposed design.

## 6. Experimental Results

The dual-mass silicon micro-gyroscopes were vacuum sealed with ceramic packages after micro-fabrication. Off-line quadrature compensation was carried out on a silicon micro-gyroscope prototype to verify the correctness of the previous design and analyses.

In the process of off-line quadrature compensation, the drive circuit worked normally. That is, in the drive direction, the two Coriolis mass were actuated into resonant vibration in anti-phase mode with the same constant amplitude. With no input angular rate, the drive-mode velocity signal and the sense-mode position signal were both extracted. In this condition, the sense-mode position signal was dominated by the quadrature vibration response y_q_. Hence, after measuring the amplitude of the sense-mode position, with Equation (9) and the scale factor of the silicon micro-gyroscope, the value of the quadrature equivalent angular rate Ω_q_ was estimated. Then with Equation (26), the needed DC voltages were calculated theoretically. Meanwhile, through the comparison of the phase information between the drive-mode velocity signal and the sense-mode position signal, the sign of the mechanical coupling stiffness was obtained. According to the needed sign of the electrostatic coupling stiffness, proper pattern groups were chosen to apply the theoretical compensation voltages. After a fine regulation of the applied DC voltages, off-line quadrature compensation was realized.

The experimental results of a silicon micro-gyroscope prototype showing quite large quadrature vibration response are presented here. The natural frequency of its drive mode is about 3.8 kHz. The value of its quadrature equivalent angular rate Ω_q_ was estimated to be 357 °/s. The sign of its mechanical coupling stiffness was found to be negative. Therefore, with Equation (26), the theoretical compensation voltages were calculated to be |V_1_^2^ − V_2_^2^| ≈ 118.6 V^2^. When the voltage loading method of single group loading was employed, Group-1 was needed to be applied with a DC voltage of V_1_ ≈ 10.89 V theoretically. When the voltage loading method of double group loading was employed, with a preset bias voltage of V_D_ = 10 V applied on both Group-1 and Group-2, the needed regulation voltage would be V_q_ ≈ + 2.97 V theoretically. The two voltage loading methods were both carried out and the experimental data of the relationships between the output amplitude of the sense-mode position signal and the applied DC voltages were obtained. At the same time, the theoretical curves of the relationships between the amplitude of the quadrature vibration response and the applied DC voltages were calculated from Equations (36) and (37).

**Figure 8.**
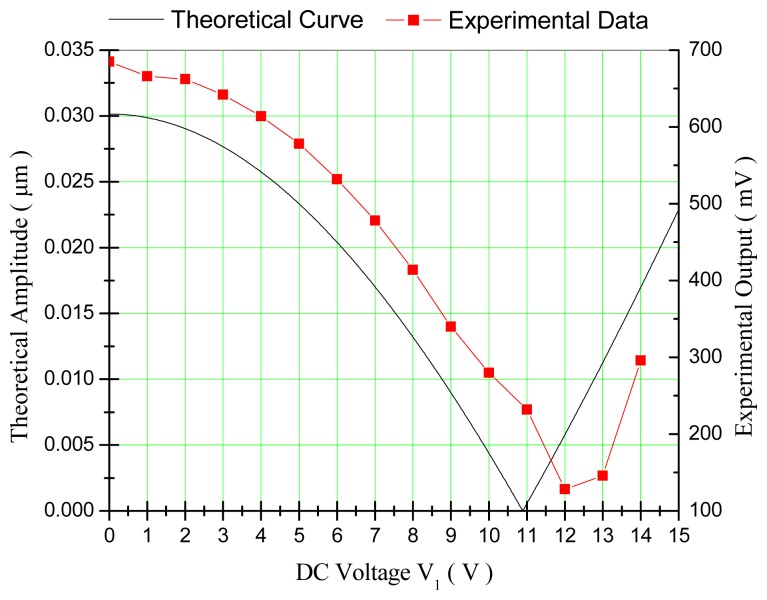
Relationships between the quadrature signal and the compensation voltage when the method of single group loading was employed.

When the voltage loading method of single group loading was employed, the comparison of the experimental data and the theoretical curve was shown in Figure 8. It can be found that the change tendency of the experimental data matched well with that of the theoretical curve. In the process of quadrature compensation, the quadrature signal has a non-linear relationship with the compensation voltage V_1_. The actually needed DC voltage for quadrature compensation was V_1_ ≈ 12 V, which is a little larger than the theoretical value.

**Figure 9.**
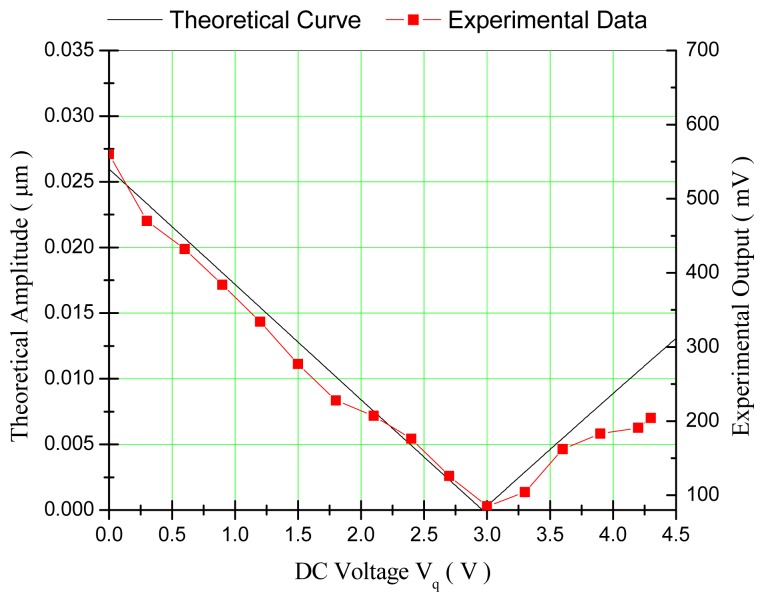
Relationships between the quadrature signal and the compensation voltage when the method of double group loading was employed.

When the voltage loading method of double group loading was employed, the comparison of the experimental data and the theoretical curve was shown in Figure 9. It can be found that the change tendency of the experimental data also matched well with that of the theoretical curve. In the process of quadrature compensation, the quadrature signal has an approximately linear relationship with the compensation voltage V_q_. The actually needed DC voltage for quadrature compensation was V_q_ ≈ 3 V, which is also a little larger than the theoretical value.

**Figure 10.**
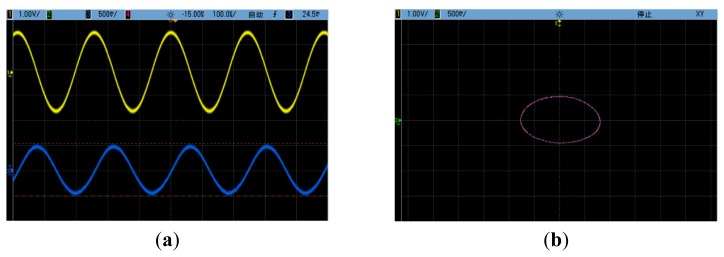
(**a**) The drive-mode velocity signal and the sense-mode position signal before quadrature compensation; (**b**) The Lissajous figure before quadrature compensation.

**Figure 11.**
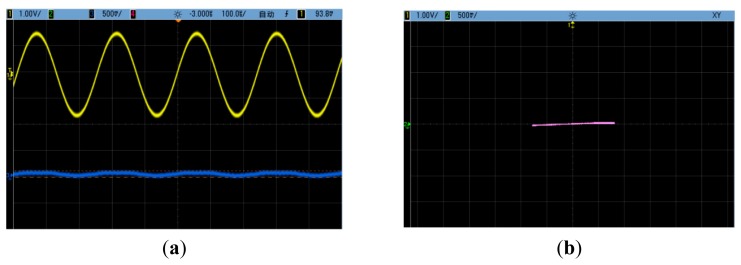
(**a**) The drive-mode velocity signal and the sense-mode position signal after quadrature compensation; (**b**) The Lissajous figure after quadrature compensation.

The drive-mode velocity and sense-mode position signals before off-line quadrature compensation are shown in Figure 10a, in which the upper wave is the drive-mode velocity and the lower one is the sense-mode position. The Lissajous figure before off-line quadrature compensation is shown in Figure 10b, in which the X-channel input is the drive-mode velocity and the Y-channel input is the sense-mode position. The two corresponding figures after off-line quadrature compensation are shown in Figure 11a and b.

It is obvious that the sense-mode position signal has the same frequency as the drive-mode velocity signal. Before off-line quadrature compensation, it was dominated by the quadrature vibration response and had an approximately 90° phase lag with the drive-mode velocity signal. After off-line quadrature compensation, the quadrature vibration response was cancelled out. However, it could be seen that a residual sense-mode position signal still existed. Its amplitude was about 100 mV in the tested silicon micro-gyroscope prototype. This residual signal had the same phase with the drive-mode velocity signal and is commonly named as in-phase error. It is mainly caused by other mechanisms, e.g. the viscous coupling between drive mode and sense mode.

## 7. Conclusions

Quadrature reduction is one of the primary issues in the design of high-performance silicon micro-gyroscopes. Among the various quadrature reduction methods, system stiffness matrix diagonalization has its peculiar advantages. In this method, with purposefully designed quadrature compensation patterns and properly applied DC voltages, an electrostatic coupling stiffness is electromechanically constructed to cancel out the mechanical coupling stiffness which is the main cause of the quadrature vibration response. This paper focus on the detailed design issues of this method and presents a complete design and application case.

For bulk silicon micro-gyroscopes, the quadrature compensation patterns which consist of comb capacitors are more suitable than those consisting of parallel plate capacitors because they provide a better overall compensation capability. There are four types of basic structural units in the mechanical design of quadrature compensation patterns. With an applied DC voltage, they can generate negative or positive dynamic electrostatic forces in sense direction. These dynamic forces are proportional to the drive-mode position, hence the needed positive or negative electrostatic coupling stiffness can be provided. Besides the useful dynamic forces, the basic structure units also generate additional static forces and torques which may cause a disturbance. A novel layout design was proposed to solve this problem. The basic structural units distributed at different places can provide same-directional dynamic forces, opposite-directional static forces and opposite-directional static torques. The opposite-directional static forces were designed to have the same absolute value. Hence no net static force exists. The opposite-directional static torques were also made to have the same absolute value. Hence no net static torque exists. When the available values of the applied DC voltages are limited, an optimization of the structural parameters, especially the gap ratio, can help improving the quadrature compensation capability.

In the process of quadrature compensation, there are two voltage loading methods: single group loading and double group loading. Single group loading is more suitable for off-line quadrature compensation. When it is employed, the quadrature signal has a non-linear relationship with the compensation voltage in the compensation process. The natural frequency of the sense mode is directly affected during the regulation of the compensation voltage. Double group loading is more suitable for on-line quadrature compensation. When it is employed, the quadrature signal has an approximately linear relationship with the compensation voltage in the compensation process. The influence of the compensation voltage regulation on the natural frequency of the sense mode can be alleviated.

The proposed design of quadrature compensation patterns was applied on an actual dual-mass silicon micro-gyroscope developed in our laboratory. With the DC voltage applied on a single pattern group no more than 12 V, the theoretical compensation capability of a quadrature equivalent angular rate no more than 412 °/s was designed. In the experiments carried out on a packaged micro-gyroscope prototype, an actual quadrature equivalent angular rate of 357 °/s was compensated. The values of the actual compensation voltages were a little larger than the theoretical ones. The correctness of the design and the theoretical analyses was verified. They can be commonly used in planar linear vibratory silicon micro-gyroscopes for quadrature compensation purpose.

## Figures and Tables

**Table 1.** Design parameters of the dual-mass silicon micro-gyroscope with quadrature compensation patterns.

**Symbols**	**Descriptions**	**Design Values**
m_c_	Coriolis mass	0.5 × 10^−6^ Kg
L_md_	Lever arm of the basic structural units in the middle part	150 μm
L_sd_	Lever arm of the basic structural units at the side parts	1200 μm
n_md_	Number of the basic structural units in the middle part	40
n_sd_	Number of the basic structural units at the side parts	5
h_0_	Thickness of the structure layer	60 μm
d_0_	Initial gap of the comb capacitors	4 μm
p	Gap ratio of the comb capacitors	2.5

## References

[1] Saukoski M., Aaltonen L., Halonen K.A.I. (2007). Zero-rate output and quadrature compensation in vibratory MEMS gyroscopes. IEEE Sens. J..

[2] Acar C., Shkel A. (2009). MEMS Vibratory Gyroscopes: Structural Approaches to Improve Robustness.

[3] Clark W.A., Howe R.T., Horowitz R. Surface micromachined z-axis vibratory rate gyroscope.

[4] Chaumet B., Leverrier B., Rougeot C., Bouyat S. A new silicon tuning fork gyroscope for aerospace applications.

[5] Zaman M.F., Sharma A., Hao Z., Ayazi F. (2008). A mode-matched silicon-yaw tuning-fork gyroscope with subdegree-per-hour Allan deviation bias instability. J. Microelectromech. Syst..

[6] Sharma A., Zaman M.F., Ayazi F. (2009). A sub-0.2°/hr bias drift micromechanical silicon gyroscope with automatic CMOS mode-matching. IEEE J. Solid-State Circuits.

[7] Tatar E., Alper S.E., Akin T. (2012). Quadrature-error compensation and corresponding effects on the performance of fully decoupled MEMS gyroscopes. J. Microelectromech. Syst..

